# Developing a System of Health Support for Young People Experiencing First-Episode Psychosis: Protocol for a Co-design Process

**DOI:** 10.2196/44980

**Published:** 2023-05-02

**Authors:** Matthew Jenkins, Tracey Gardiner, Crystal Pekepo, Pāyal Ramritu, Briony Drysdale, Susanna Every-Palmer, Victoria Chinn

**Affiliations:** 1 Department of Psychological Medicine University of Otago Wellington Wellington / Te Whanganui-a-Tara New Zealand; 2 Toi Tangata Auckland / Tamaki Makaurau New Zealand; 3 School of Health Te Herenga Waka Victoria University of Wellington Wellington / Te Whanganui-a-Tara New Zealand

**Keywords:** psychosis, health, well-being, co-design, lived experience, early intervention

## Abstract

**Background:**

People living with psychosis face a substantially increased risk of poor psychological well-being and physical health and premature mortality. Encouraging positive health behaviors from an early stage is crucial to the health and well-being of this population but is often overshadowed by symptom management within early intervention services.

**Objective:**

Experience-based co-design is a participant-centered approach that aims to combine service user narratives with service design methods to design systems of support for health and well-being. This study aims to use experience-based co-design principles to co-design a system that supports the health and well-being of young people experiencing first-episode psychosis (FEP), which considers the lived experience of these people within the context of early intervention services. We also aim to develop a set of principles to guide future systems to support the health and well-being of young people experiencing FEP.

**Methods:**

Up to 15 young people living with FEP aged 16 to 24 years who are service users of early intervention services in psychosis, their immediate support networks (family or friends), and health professionals involved with early intervention services in psychosis will be invited to participate in a series of co-design workshops. Data will be collected in various forms, including expressive forms (eg, art and spoken word) and traditional methods (interview transcription and surveys), with phenomenographic and thematic analyses being used to understand these data. Furthermore, the co-design process will draw upon indigenous (Māori) knowledge and the lived experience of mental health services from the perspectives of the members of the research team. The co-design process will be evaluated in terms of acceptability from the perspective of service users via rating scales and interviews. The study will be conducted within the Lower North Island in Aotearoa New Zealand.

**Results:**

Data collection will be performed between August 2022 and February 2023. Drawing from extended consultations with service users and service providers, we have developed a robust co-design process with which we intend to collect rich qualitative and quantitative data. The results of this process will be used to create a system of support that can be immediately applied and as preliminary evidence for funding and resource applications to deliver and evaluate a “full” version of the co-designed system of support.

**Conclusions:**

The co-designed system of support and accompanying set of principles will offer a potentially impactful health and well-being intervention for young people experiencing FEP in Aotearoa New Zealand. Furthermore, making the co-design process transparent will further the field in terms of providing a blueprint for this form of participant-focused research.

**Trial Registration:**

Australian New Zealand Clinical Trials Registry (ANZCTR) ACTRN12622001323718; https://www.anzctr.org.au/Trial/Registration/TrialReview.aspx?id=384775&isReview=true

**International Registered Report Identifier (IRRID):**

DERR1-10.2196/44980

## Introduction

### Background

The physical health of people living with serious mental illness is worse than that of the general population [1]. Cardiovascular disease is more prevalent in this population, and life expectancies are shortened on average by 25 years, a sobering statistic described as the “scandal of premature mortality” [2]. Ultimately, serious mental illness, particularly psychosis, has been identified as a significant independent risk factor for poor cardiovascular health and increased mortality [3]. Although antipsychotic medication has been implicated in the elevated risk of cardiovascular disease [4-6], modifiable risk factors such as smoking, substance use, poor diet, and insufficient physical activity also play an important role. The relationship between mental illness and poor physical health highlights the holistic nature of health such that physical and mental aspects (among other equally important parts such as social and spiritual well-being) are deeply interconnected [7]. Failure to support the health of people living with psychosis has led to international calls for interventions that prioritize both physical and mental health, with health behaviors as a key point of leverage [8].

The timing of these interventions is crucial. There is a critical window of opportunity to provide support for young people experiencing their first-episode psychosis (FEP) in maintaining positive health behaviors [9]. However, in a review of lifestyle interventions for people experiencing FEP, Gates et al [10] highlighted the lack of such programs, and even less are those programs that are based on robust evidence and theory in Aotearoa New Zealand (NZ). A lack of support for the physical health of people experiencing psychosis has been noted as a major source of inequity [1].

The long-term effectiveness of any health intervention depends on its feasibility within a given health service, which itself relies on the uptake and engagement by the target group and those who support them (eg, family and carers). A major factor in the acceptability of a lifestyle intervention is the extent to which it is suitably adapted to the specific population. People experiencing FEP may encounter barriers to health behaviors related to antipsychotic medication (eg, metabolic issues and effects on energy levels), psychological factors (eg, motivation and self-efficacy), stress, and a lack of support [11,12]. They are also likely to prioritize their own goals over those imposed on them by health services. Therefore, elucidating and addressing such barriers and understanding the goals of people experiencing FEP are crucial for intervention success.

Valuing lived experience positions service users as “experts by experience.” As such, the personal experience of living with mental distress and using mental health services provides unique and essential insight into how to best serve the users of these services. Early and authentic involvement with people with lived experience can lead to enhanced credibility, quality, and relevance of services and to a greater sense of empowerment and agency for the service users involved in the development process [13-15]. Working alongside experts by experience is increasingly recognized as being crucial to service development, both within the mental health sector [13,14] and specifically with people experiencing psychosis [15].

Support from service providers is also necessary for the long-term sustainability of a new health intervention [16]. Frontline providers are responsible for implementing the intervention beyond the life of the project, as well as directly influencing the extent to which participants change their behaviors [17]. Therefore, when developing an intervention, we must understand how the intended intervention outcomes align with providers’ service objectives, as well as the extent to which they can be adequately resourced and implemented in practical terms. By addressing these factors, we increase the likelihood of ongoing intervention success by encouraging service providers to “buy-in” [16].

Co-designing interventions alongside participants and service providers effectively optimizes intervention feasibility, uptake, and sustainability [18,19]. An additional benefit of co-design is that it supports participants’ self-determination by providing them with an opportunity to exercise autonomy in terms of their physical health care [20]. To date, co-designed interventions that support the physical health of people experiencing FEP have been missing in Aotearoa NZ [1]. This research responds to global calls to develop such interventions [8] while aligning with localized health service contexts.

### Research Objectives

#### Obtain Narratives Regarding the Lived Experience of Young People Being Healthy During FEP

How do young people experiencing FEP define health (ie, what “health” looks like and what components contribute to a healthy life)? What are the key barriers and facilitators to achieving positive health? What is missing in the current health care system or community that can support a healthy life?

#### Develop a System of Support for Leading a Healthy Life

Alongside service users, their family or support people and service providers cocreate a system of support, including specified components (eg, physical activity sessions or access to peer support groups) and format (eg, in-person or web-based connecting events).

#### Create a Set of Principles to Guide Early Intervention Health Care

Using data obtained across all stages of the co-design process, we aim to distill a set of principles that participants think should guide the support of young people experiencing FEP. For example, such principles might include collaboration, social connections, action, listening, or autonomy.

#### Devise a Strategy for the Future Delivery of a System of Support

Furthermore, we aim to obtain an understanding of the resources that are needed to deliver the components that have emerged from the co-design process and to create a plan to obtain these resources, whether internally from the existing health care system or externally from community, project, or research grants.

## Methods

### Design

An experience-based co-design (EBCD) approach [18] will be used to understand the lived experiences of service users experiencing FEP and to design an effective system of support for service users of early intervention services in psychosis (EISP). An iterative approach is an important aspect of EBCD because it allows for the reflection and hypothesis testing of prototype solutions [21].

The co-design process will include five key phases:

1. Contextual inquiry (understanding the context, including issues faced by participants and contextual constraints).
2. Participatory design (working with service users and service providers to identify initial solutions).
3. Product design (working with the service provision advisory group to develop a support system based on participatory design outcomes).
4. Prototype as hypothesis (asking service users to approve or provide comment on the developed system of support).
5. Final program revision.

For the purpose of this project, contextual inquiry and participatory design will be combined into one phase. In addition, a pre–co-design focus group with prospective service user participants is used to design the co-design workshops. Following these phases, the co-designed system of support will be presented to all stakeholders, and an audit will be conducted to identify the resources required to deliver and evaluate the system of support. Service users, their close supporters (eg, family members and other support people), and staff within the EISP will be consulted via a combination of interviews and focus groups across these phases. Involving service users and their support group in the design process will provide them with opportunities to exercise autonomy in their own health care, while service providers will offer critical insights regarding the intervention feasibility and will support future implementation success.

**Figure 1:**
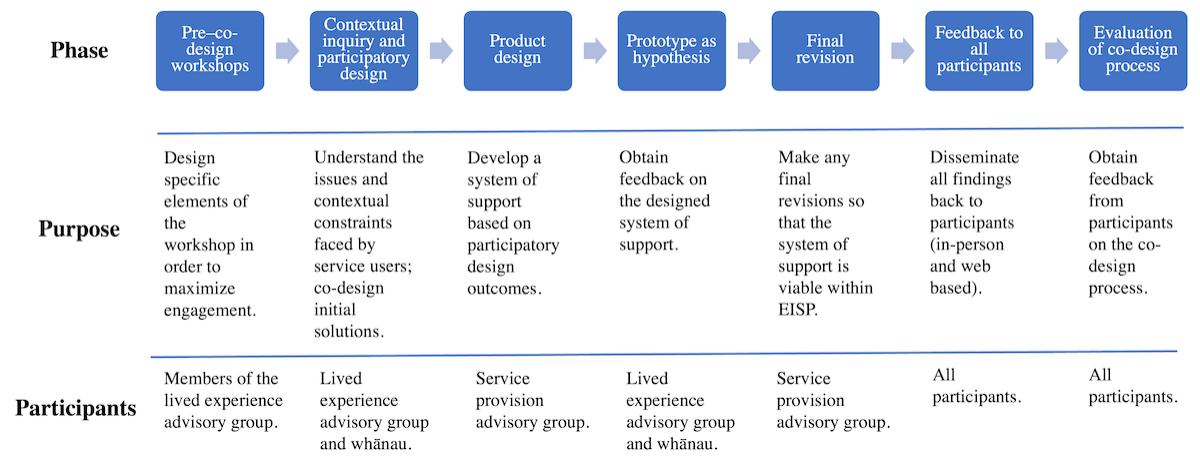
Overview of the co-design process.

An overview of the co-design process and the involvement of the participants at different stages are summarized in Figure 1.

### Context

Critical to conducting research in Aotearoa NZ is the duty to uphold Te Tiriti o Waitangi (the Māori version of the Treaty of Waitangi), which recognizes shared governance between the Indigenous people of Aotearoa NZ and the British Crown as well as a responsibility for supporting Māori health. Failure to do so in the past is exemplified by the overrepresentation of Māori with FEP and psychosis [22]. This co-design project works in partnership with a Māori agency, Toi Tangata, to ensure that the project is informed by mātauranga Māori (Māori knowledge) so that the resulting system of support is culturally responsive and safe for Māori with FEP in Aotearoa NZ.

Furthermore, this project takes place against a background of change to Aotearoa NZ health service delivery. This change involves the dissolution of 20 district health boards into a larger unifying health board and the establishment of an independent Māori Health Authority. Relevant to this project, the reform has been identified as a catalyst for change toward strengthening primary health care contextualized to local communities and improving the way services are used and accessed [23].

### Adopting a Māori Lens for Co-design

**Figure 2:**
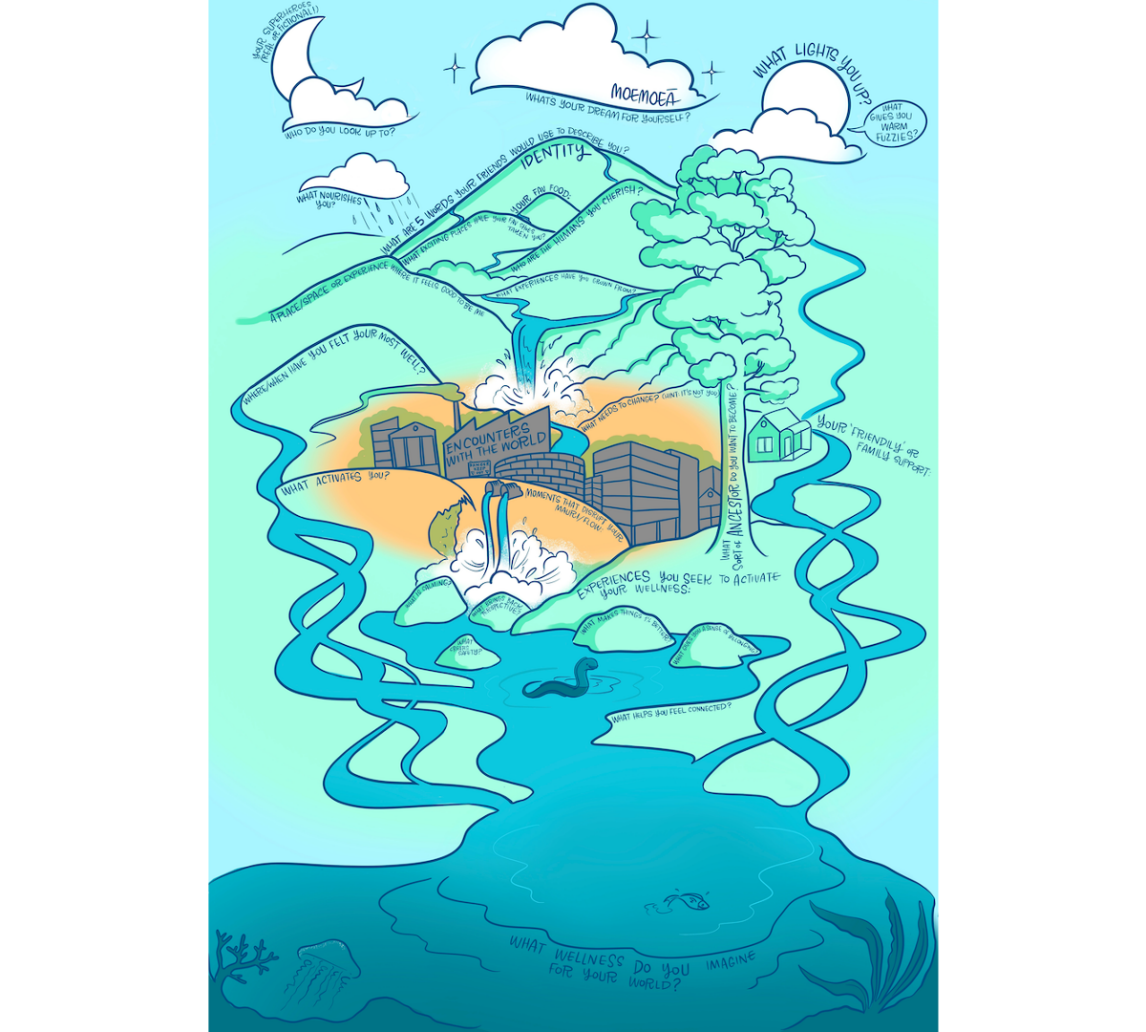
“Navigating the Puna” framework used to guide contextual inquiry. Translations from Māori to English: “Moemoeā” is often used to mean “dreams and aspirations”; “mauri” often refers to one’s “life force or essence”. Copyright League of Live Illustrators, New Zealand.

This project embeds Māori principles, which enable approaches that are engaging and creative. For example, a key principle is the process of whakawhanaungatanga (relationship building) to create a space in which participants feel safe to tell their stories and narratives. Indeed, this narrative storytelling aspect is another key principle of Māori-based approaches, with pūrākau (stories) relating to one’s own individual experiences being an important way to create meaning. The key features of this project that align with this approach are the use of water (wai) as a metaphor (Figure 2) and allowing participants to tell their story in different creative ways so that they are understood by their peers and researchers (Multimedia Appendix 1).

Furthermore, this research is guided by the Māori model of health, Te Whare Tapa Whā, that conceptualizes health as a whare (house) with its stability dependent on the 4 walls of well-being: tinana (physical), hinengaro (mental), wairua (spiritual), and whānau (social) health [7]. This model is inherently holistic compared with typical Western-influenced models, such that it acknowledges the importance of the dynamic relationship between the many dimensions of well-being. This holistic approach has the potential to benefit all clients of health services, including those who identify as Māori. Embedding Te Whare Tapa Whā and Māori principles into the project process and design is a strengths-based approach to incorporating mātauranga Māori into health care, rather than solely focusing on Māori health deficits.

### Research Team and Partners

Researchers from the University of Otago and Te Herenga Waka Victoria University of Wellington are coleading this project. Other team members include a project manager and 3 early career researchers employed as research assistants or assistant research fellows. Throughout the research team, there are varying degrees of lived experiences of FEP and mental distress. Given the existing discrimination of people living with mental illness or distress in employment (both perceived and objective) [24,25], this was an opportunity to increase the research capacity within the workforce in terms of early career researchers with lived experience of FEP and mental distress, as well as to bring in crucial lived experience perspectives to the research process.

The Māori health agency Toi Tangata has codeveloped the co-design process alongside the research team and will cofacilitate the workshops. In addition, the League of Live Illustrators will be embedded in some workshops to provide live interactive note-taking in the form of illustrations. Partners within the EISP will be project facilitators and assist with participant recruitment (service users, family members, and service providers).

A steering group will guide project decision-making from procedural and policy perspectives to maximize project impact. Steering group members include clinical and academic psychiatrists, representatives of nongovernment advocacy organizations, and both Māori and non-Māori public health researchers.

### Participants

#### Lived Experience Advisory Group

This is the primary participant group. The community of young people experiencing FEP is heterogeneous in terms of cultural background, gender, and education. The research team will work closely with the EISP to identify participants within their services who are representative of these demographic characteristics.

#### Whānau

Recognizing the wider network of people who support young people experiencing FEP, we will seek the perspectives from service users’ whānau. Whānau is a Māori word that is often translated to “family members”; however, whānau can also refer more widely to close family friends or people who support another person. Therefore, the word whānau is used throughout this manuscript to refer to both family members and people who support service users (in a nonprofessional capacity).

#### Service Provision Advisory Group

The service provision advisory group will consist of health professionals who support service users in some professional or voluntary capacity, which may include psychiatrists, clinical psychologists, occupational therapists, and pharmacists, who have experience in treating, managing, and supporting young people experiencing FEP. It is anticipated that they will mostly be working within the EISP (and therefore have an internal understanding of mental health services), but participation will also be open to people working in an adjacent supportive capacity (eg, nutritionists, physical activity providers, and community support workers).

The iterative procedure means that these groups will be consulted at least twice during the co-design process.

### Recruitment

For the lived experience advisory group, study information will be relayed to EISP service users via posters, email, newsletters, and caseworkers working within EISP. In terms of sample size, approximately 125 clients are engaged with early intervention FEP services in the target catchment area. We aim to recruit up to 15 clients from this pool.

For the whānau workshop, members of the lived experience advisory group will be asked to identify their supporters and pass on the study information to them. We aim to recruit 1 whānau member per participant in the lived experience advisory group.

The project team will recruit service provision advisory group members through their existing networks and local early intervention symposia. Study information will also be circulated to relevant experienced health professionals via EISP staff team meetings, newsletters, and email.

### Participant Remuneration

Our group recognizes that a co-design process that truly values the opinions and narratives of service users and their whānau should compensate them in a way that recognizes them as experts in their own health care. As such, members of the lived experience advisory group will receive NZ $100 (US $62) in vouchers as compensation for their time. In addition, these participants will be offered a choice of a framed print or tote bag depicting an illustration produced during the first workshop. Whānau participants will receive NZ $50 (US $31) for attending the single 2-hour workshop.

Members of the service provision advisory group who are employees of mental health services will not be remunerated, as participation is interpreted as part of their employment. Any members of the service provision advisory group who are engaged as volunteers and not in a professional capacity will be offered remuneration in line with service users and their whānau.

### Co-design Workshop Format

#### Overview

The workshop format was developed in collaboration with members of the lived experience advisory group, the research team, and partners over a series of meetings. Together, this group contained expertise in the lived experience of psychosis and EISP, community engagement, mātauranga Māori, and co-design. A key objective in designing these workshops was to ensure that as many participants as possible have the opportunity to engage meaningfully with the project.

Two key decisions in the design were whether the workshops should include all participants (ie, service users, whānau, and service providers) or whether they should be separated into 3 distinct workshops and where the workshops should be held (ie, in-person or web-based workshops and specific location if in person).

#### Combined or Separated Groups

The group decided that the lived experience advisory group, their whānau members, and the service provision advisory group would each attend separate workshops, thus enabling frank and open discussions regarding service users’ health and related barriers and not being inhibited by the presence of another group. However, the lived experience advisory group will be given the option of being accompanied by a whānau member or caseworker for support at each workshop they attend. One-to-one interviews (either web-based or in-person interviews) will be offered to members of the lived experience advisory group who are uncomfortable speaking within group situations or unable to attend workshop times.

#### Platform and Location

With regard to choosing web-based versus in-person workshops, members of the lived experience advisory group indicated a preference for in-person workshops to maximize engagement and relationship building. Feedback regarding whānau members was that they were often employed full time and had time and travel constraints. Therefore, whānau members will be invited to a web-based evening workshop during the contextual inquiry and participatory design phases.

Many members of the service provision advisory group are health professionals with time constraints in terms of their clinical duties. For workshops involving this group, in-person attendance will be offered for the first relevant session (product design) to create initial engagement and investment in the project. The second round of sessions involving the advisory group (product revision) will be offered as a web-based workshop and survey.

The venue chosen for the co-design and similar workshops involving service users is important in terms of setting participant expectations and creating a sense of security and willingness to share experiences. The research team considered the balance between being in a familiar space that was well known to service users (ie, EISP clinical services) and the need for a space that was not directly associated with a clinical setting, thus increasing the likelihood that participants would feel free to share their experiences (ie, in a neutral venue). It was decided that rooms at the University of Otago campus will be used for the first workshop involving the lived experience advisory group. For whānau, this did not apply because the workshop will be held on the web. For the advisory group, the venue will be EISP clinical service centers.

#### Workshop Facilitation

The workshops will be cofacilitated by diverse members of the project team across all the co-design phases. All facilitators have significant experience in facilitating workshops in community settings, including with young people experiencing FEP, whānau members, and service providers. Notably, several facilitators have lived experience of FEP and mental distress. This will create an opportunity for positive role modeling and increased the relatability of the workshop content. The workshop design draws from contact-based antistigma and discrimination intervention models, which have been used to guide efforts to counter stigma toward mental distress in Aotearoa NZ [26,27].

Although lived experience–directed antistigma interventions typically target groups who hold the power to discriminate, the model here also functions to alleviate internalized stigma for participants who experienced FEP. The power of the contact model [27] draws on the principles of all group members having equal status and providing the opportunity for relationship building, active cooperation, and the pursuit of a mutual goal. Other members of the facilitation team have extensive knowledge and experience in incorporating the Māori worldview into co-design processes.

### Ethics Approval

The project has been approved by the University of Otago Health Ethics Committee (ref H22/048) and has undergone a Māori consultation in line with guidelines pertaining to research based in Aotearoa NZ. Considering that workshops may be used to address potentially sensitive health-based topics and thus present a small risk of psychological distress, an experienced clinical psychologist (not directly associated with EISP) will also attend sessions involving the lived experience advisory group to meet the ethical guidelines regarding psychological safety. The project has also been registered with the Australian NZ Clinical Trials Registry ACTRN12622001323718.

## Results

Details of the co-design workshops are summarized in Multimedia Appendix 1.

### Phase 1a: Contextual Inquiry and Participatory Design With the Lived Experience Advisory Group

The illustrated framework (“Navigating the Puna”; Figure 2) was developed by research team members with lived experience of mental distress, the Māori co-design partners (Toi Tangata), and artists from the League of Live Illustrators. The illustration encompasses the main objectives of contextual inquiry and participatory design (eg, experiences of health, barriers to and facilitators of health and well-being, and how health and well-being can be supported). “Navigating the Puna” is based on mātauranga Māori in that the puna (fountain or spring) represents the person with a lived experience of FEP. The illustration will be used as a framework to guide the first workshop with the lived experience advisory group (workshop 1a).

Contextual inquiry with the lived experience advisory group will seek to understand (1) how young people conceptualize health while living with FEP, (2) what constitutes a healthy life and the barriers and facilitators to leading a healthy life, and (3) the contextual constraints they encounter.

A phenomenographic approach will be used to provide participants in the lived experience advisory group the opportunity to express their experiences in a way that is meaningful to them and not constrained to typical focus group–style data collection. Phenomenography is “a qualitative research approach used to describe variations in people’s experiences through their own discourse, not to derive general principles of how things appear but to make known the way(s) experiences can be commonly understood” [28], with the goal of discovering different ways of understanding the experience of phenomena [29-31].

In line with this approach, participants will be offered several platforms to describe their experiences. Opportunities will include writing or audio recording their experiences in the form of stories, simple descriptions, or voice notes. A data wall will be set up to which participants can contribute post-it notes at any point during the workshop as ideas occur. Alternative creative outputs include drawings, paintings, poetry, and clay sculptures. There has been an increasing trend in using creative and art-based approaches in health care, for example, photovoice, visual artistic methods such as drawing and painting, and literary arts such as poetry and storytelling [32-39]. All these are accepted approaches for collecting phenomenographic data [29].

When possible, photos of creative outputs will be taken, and participants will be asked to provide a brief description of the output in their own words to aid later interpretation and analysis. Members of the research team will also collect data through a process of interactive note-taking using a whiteboard that will be visible to the participants throughout the day. These notes will be collated and photographed at the end of the workshop.

Data will be analyzed using an approach aligned with both thematic analysis [40] and phenomenographic analysis [41]. These approaches share a common process that is characterized by several stages: (1) familiarization (data are reviewed taking note of initial ideas); (2) identification (data that are related to the phenomenon being described are identified); (3) sorting (identified data are organized into “pools of meaning” according to similarities); (4) contrasting and categorizing (themes are contrasted, and categories are generated with descriptions); and (5) reliability checking (a portion of the data coded by independent researchers will be checked for intercoder reliability).

The result will be a collection of themes regarding service users’ experiences of health and well-being while living with FEP and facilitators of and barriers to leading healthy lives.

For the participatory design aspect of the workshops (for both 1a and 1b), ideas with regard to possible program components, features, or structures that participants create will be noted, collated, and taken through to phase 2 with the service provision advisory group.

### Phase 1b: Contextual Inquiry With Whānau

A web-based focus group will be held with whānau to explore their experiences of supporting a young person living with FEP and their perspectives on what additional supports are required from health care services. This focus group will be audio recorded and analyzed using thematic analysis as described in the previous section. The findings from phase 1b will supplement the themes identified from the lived experience advisory group.

### Phase 2: Product Design With Service Provision Advisory Group

An in-person focus group will be held, and the sessions will be audio recorded. Key areas that will be addressed include participants’ experiences in supporting young people living with FEP in terms of maximizing health and well-being, perceived barriers to and facilitators of positive health behaviors, and any other aspects that they see as being relevant.

A summary of findings from the previous workshops with the lived experience advisory group and their identified whānau will be presented. Participants will be asked to respond to the proposed solutions to ascertain which features and components they see as feasible in the context of the existing EISP and which of these may be feasible in the future. Participants will be asked to identify other potential approaches to supporting health and well-being that align with the initial design solutions proposed by the lived experience advisory group.

The research team will collate these responses and integrate them with the initial co-designed system of support from the previous workshops, resulting in a “prototype” support system or program.

### Phase 3: Prototype as Hypothesis With the Lived Experience Advisory Group

A web-based focus group with the lived experience advisory group will be held, and sessions will be audio recorded. The prototype will be presented to participants, who will be asked to provide feedback in terms of the acceptability and likelihood that they would use it. Participants will also be asked to identify any issues that they foresee with the prototype and any revisions to overcome such issues. Any omissions in terms of requested features, components, or formats will be explained.

The research team will collate these responses and integrate them to create a new revised version of the system of support or program.

### Phase 4: Final Revision With the Service Provision Advisory Group

Feedback will be sought on the revised version of the system of support or program in the form of a web-based survey. The system or program is presented in full as a web-based static document, and the feedback sought will pertain to the feasibility of the various features in the form of 1 to 10 rating scales. For features that are deemed unfeasible, participants will be asked to provide a rationale for this. Participants will be able to leave open-text feedback on each feature or program as a whole.

### Post–Co-design Reflection and Presentation of Findings

The project team will produce a final version of the system or program that will account for the data and feedback offered by all participants. This will be presented to all participants and invited guests during a seminar.

### Evaluation of the Co-design Process

Members of the lived experience advisory group will be asked to provide feedback on the co-design process. Feedback will be sought at the final program presentation, with an option to provide web-based feedback. Specifically, feedback will be sought regarding (1) the extent to which participants felt like their autonomy was supported during the co-design process, (2) the extent to which participants came away with a sense of hope for their own lives and for their health care, (3) whether participants felt listened to throughout the process, and (4) to what extent participants feel confident that change might happen as a result of the co-design workshop.

This feedback will be collected using rating scales (eg, “On a scale of one to ten, how much confidence do you have that change might occur as a result of this co-design process?”).

Regarding autonomy and feelings listened to, the Healthcare Climate Questionnaire (HCCQ) will be used. The HCCQ also contains questions pertaining to perceptions of being listened to by facilitators (eg, “The workshop facilitators listened to how I would like to do things regarding my health”).

### Postproject Audit

The research team will work with project facilitators at the EISP and health care managers to identify a minimal deliverable version of the system or program and if necessary, identify potential sources of funding to deliver and evaluate a “full” version of the system or program (eg, research grants, internal health care funding, and Ministry of Health grants). This audit will take the form of a discussion group and feedback via e-mails.

## Discussion

### Overview

This project has four objectives: (1) to understand the lived experiences of young people in being healthy during FEP, (2) to develop a system of support for leading a healthy life, (3) to create a set of principles that guide early intervention health care for young people experiencing psychosis, and (4) to devise a strategy for the future delivery of a system of support for health and well-being within this community. To achieve these objectives, an EBCD approach will be undertaken, within which we will work alongside and listen to key stakeholders (service users with lived experiences, their whānau, and service providers) through a series of workshops. The results of this project will provide us with (1) a richer understanding of the lived experiences of young people living with FEP, (2) a co-designed system of support for health and well-being, (3) a set of guiding principles for developing such systems of support, and (4) a clear strategy for delivering and evaluating the co-designed system.

This co-design project responds to the lack of interventions aimed at supporting the health of young people experiencing FEP [8]. Close collaboration with experts in the lived experience of FEP and mātauranga Māori works to ensure the relevance of, and engagement with, the co-design process and the resulting support system by end users in Aotearoa NZ. This approach answers the calls for health care research and services to genuinely engage with indigenous (Māori) knowledge systems in a collaborative rather than prescriptive and tokenistic manner [42]. This collaborative approach also works alongside EISP service providers to support the longevity of the end product beyond the life of the project.

### Limitations

This project is specific to the context of Aotearoa NZ. Although it provides a useful example of how to engage with young people with lived experiences of FEP, Indigenous stakeholders, and EISP providers, careful consideration is needed when working outside of this specific context. In addition, the authors acknowledge that young people experiencing FEP engage in a wide variety of services. This project is co-designed specifically with young people at the stage of FEP and with EISP providers. Therefore, reproducing this study with other services and with persons at a different stage of diagnosis may likely produce a different result. Given that physical health inequities are experienced by people with serious mental illnesses in broader terms [8], further research is needed to investigate how this approach may be applied to people with other diagnoses and service providers.

A further limitation is that although the power of the contact model is named as a key guiding framework for the co-design process, a true representation of this model would have resulted in combined group discussions (ie, service providers, service users, and whānau). However, our priority was to create a safe space for service users to discuss, and owing to the explicit request of the service users within our consultation round, we decided to keep these stakeholder groups separate, resulting in a power of contact model adapted to suit our primary participants.

This protocol provides an example of how health professionals and researchers can engage in the co-design process toward a more collaborative way of working and participant-centered support. It is our hope that the results of this project will address the inequities that young people experiencing FEP face and improve their health and well-being. Furthermore, we hope that the guiding principles produced by this project can be applied to develop similar support systems in communities outside the area in which this research takes place.

## Appendix

Multimedia Appendix 1 Co-design workshops details.

## Abbreviations

EBCD: experience-based co-design
EISP: early intervention services in psychosis
FEP: first-episode psychosis
HCCQ: Healthcare Climate Questionnaire
NZ: New Zealand

## Data Availability

The data collected in this study will be deidentified and made openly available in Figshare [43].
