# Supplementary material for: Developing a System of Health Support for Young People Experiencing First-Episode Psychosis: Protocol for a Co-design Process
Source: JMIR Res Protoc. 2023 May 2;12:e44980. doi: 10.2196/44980 (PMC10189618; doi:10.2196/44980)
Supplement: Multimedia Appendix 1 [file resprot_v12i1e44980_app1.docx]

Appendix 1. Co-design workshops details.

| **Phase** | **Participants** | **Platform and format** | **Objective(s)** | **Specific activities** | **Form of data collection and analysis** |
| --- | --- | --- | --- | --- | --- |
| **Phase 1a:** contextual inquiry and participatory design | Lived experience advisory group | In-person workshop (four hours) or interviews (45-60 minutes)  *(participants’ choice)* | - Define what good health and wellbeing looks like, sounds like, feels like. - Understand the barriers and facilitators to leading healthy lives. - Design initial solutions and principles that can support and guide personal health and wider healthcare. - Create ownership of the programme. - Role modelling and participant engagement. | - The “Navigating the Puna: framework will be used to facilitate participants’ sharing of their lived experiences. This will involve: - group discussions; - individual written contributions (post-it notes, reflections); - creative outputs via design stations (sculptures, audio recordings, drawings, paintings, poetry). - Sharing of experiences by facilitators with lived experience of FEP. | - Alongside any output that is open to interpretation (e.g., creative outputs), participants will be asked to provide a short written or verbal description of what they have created. - Research assistants will take live notes and will create in situ summaries of sections corresponding to each workshop objective. - Phenomenographic analysis and thematic analysis of linear and creative output data (e.g., photos of visual creative outputs). - An open channel for continued feedback will be provided via email, to allow for participants to offer their perspectives following a period of post-workshop reflection. Such feedback will be admissible up to seven days following each workshop. |
| **Phase 1b:** contextual inquiry and participatory design | Whānau members | Online workshops (two hours) | - Understand the barriers and facilitators to leading healthy lives from the perspectives of whānau. | - Group discussions regarding: what is most important in your whānau members’ health care?; What barriers and facilitators to health and positive health behaviours have you observed? | - Audio recordings, which will be analyzed using thematic analysis. |
| Inter-phase analysis | The research team will conduct a preliminary thematic analysis that seeks to outline the barriers to and facilitators of health, and the wider lived experience of service users and their whānau, to be presented to service providers in the Phase 2. | | | | |
| **Phase 2:** programme design | Service provision advisory group | In-person workshop (two hours) | - Understand the feasibility of the initial suggested supports with regards to medication, symptomatology, available support within health services etc. - Create a system of support or set of features for this system. | - Present results from previous phase and prompt group discussion, specifically regarding specific system features that take into account the lived experience of service users. | - Audio recordings, which will be transcribed. - Live note-taking and data walls. |
| Inter-phase reflection | The project team will create a prototype system of support/programme that accounts for the experiences and features discussed by all three participant groups. | | | | |
| **Phase 3:** prototype as hypothesis | Lived experience advisory group. | Participants’ choice of web-based workshop (one hour) or web-based survey. | - Receive feedback on the revised programme or system of support. - Make further changes as necessary. | - The prototype will be presented and rationale presented for specific components. - *Web-based* *workshops:* focus group­style discussion regarding components of the programme or system of support. - *Web-based* *survey:* outline of the programme or system of support presented and online feedback elicited.   Participants will be asked to name the project or system of support. | - *Web-based workshops:* audio recordings of group discussion, which will be analyzed using thematic analysis. - *Web-based survey:* rating scales and open text responses regarding: - feasibility and acceptability - areas of concern - suggested revisions to the programme or system of support. |
| **Phase 4:**  final revision | Service provision advisory group | Participants’ choice of web-based workshop (one hour) or web-based survey. | Make any final revisions so that the system of support is viable within/alongside EISP. | The final system of support/programme will be presented and final revisions. | *Web-based workshops:*  Audio recordings of group discussion.  *Web-based survey:*  Rating scales and open text responses regarding any suggested final revisions to the system/programme. |
| Post-co-design reflection | Members of the project team will conduct a full thematic analysis to identify a set of high-level principles that emerged from the co-design process, which are shared amongst service users, their whānau, and service providers. These will describe the way in which health and wellbeing could/should be supported within FEP early intervention. | | | | |
| Presentation of findings | All participants and invited guests, including early intervention healthcare managers. | In-person and web-based (blended) seminar. | To present the findings of the co-design process. | The co-designed system of support and identified principles will be presented by members of the project team. | Feedback will not be directly sought, but will be welcomed. |
| Evaluation of the co-design process | Lived experience advisory group. | Web-based survey. | Understand the experiences of participants with regards to confidence in changes being made to health support, hope for the future, and confidence in their own ability to lead healthy lives. | Completion of rating scales for confidence in changes being made to health support, hope for the future, and confidence in their own ability to lead healthy lives. For perceived autonomy, the Healthcare Climate Questionnaire (HHCQ; modified version) will be used. | Quantitative data obtained from online survey |
| Post-project audit | Research team, EISP project facilitators, and healthcare managers. | In-person. | Identify a minimal deliverable version of the system, and identify potential sources of funding. for a full version of the system. | Group discussion. | Grouping of potential systems of support into *immediately* *deliverable* and *deliverable with additional resources*. |
